# Constructing segmentation method for wheat powdery mildew using deep learning

**DOI:** 10.3389/fpls.2025.1524283

**Published:** 2025-05-26

**Authors:** Hecang Zang, Congsheng Wang, Qing Zhao, Jie Zhang, Junmei Wang, Guoqing Zheng, Guoqiang Li

**Affiliations:** ^1^ Institute of Agricultural Information Technology, Henan Academy of Agricultural Sciences, Zhengzhou, China; ^2^ Huanghuaihai Key Laboratory of Intelligent Agricultural Technology, Ministry of Agriculture and Rural Areas, Zhengzhou, China; ^3^ Institute of Plant Protection Research, Henan Academy of Agricultural Sciences, Zhengzhou, China

**Keywords:** wheat powdery mildew, deep learning, Swin-Unet, SENet, ResNet

## Abstract

Powdery mildew is an important factor affecting wheat yield and global food security as well as a leading factor restricting the sustainable development of agriculture. Timely and accurate segmentation of wheat powdery mildew image is an important practical significance for disease-resistant breeding and precise control. In this study, RSE-Swin Unet was proposed based on the Swin-Unet architecture to address the complex morphology of wheat powdery mildew lesions, blurred boundaries between lesions and non-lesions, and low segmentation accuracy. The method combines ResNet and SENet to solve the abovementioned problem. Firstly, the attention mechanism module SENet is introduced into Swin-Unet, which can effectively capture global and local features in images and extract more important information about powdery mildew. Secondly, the output of the SENet module add to the corresponding feature tensor of the decoder for subsequent decoder operations. Finally, in the deep bottleneck of Swin-Unet network, ResNet network layers are used to increase the expressive power of feature. The test results showed that in the experiment with the self-built wheat powdery mildew dataset, the proposed RSE-Swin Unet method achieved MIoU, mPA, and accuracy values of 84.01%, 89.96%, and 94.20%, respectively, which were 2.77%, 3.64%, and 2.89% higher than the original Swin-Unet method. In the wheat stripe rust dataset, the proposed RSE-Swin Unet method achieved MIOU, MPA, and accuracy values of 84.91%, 90.50%, and 96.88%, respectively, which were 4.64%, 5.38%, and 2.84% higher than those of the original Swin-Unet method. Compared with other mainstream deep learning methods U-Net, PSPNet, DeepLabV3+, and Swin-Unet, the proposed RSE Swin-Unet method can detect wheat powdery mildew and stripe rust image in a challenging situation and has good computer vision processing and performance evaluation effects. The proposed method can accurately detect the image of wheat powdery mildew and has good segmentation performance, which provides important support for the identification of resistance in wheat breeding materials.

## Introduction

Wheat is a widely cultivated food crop in the world, providing staple food for approximately 40% of the global population (Tester and Langridge, 2010). With the aging of the population structure, reduction of arable land area, and impact of abnormal climate, there are many uncertainties in the formation of wheat yield (Su et al., 2023). According to FAO estimates, global crop yields are expected to decrease by 20% to 40% annually owing to pest and disease losses (Zhang et al., 2020). Wheat powder mildew is a prevalent disease worldwide. It is an airborne fungal disease caused by powdery mildew that seriously threatens global wheat yield (Bennett, 1984; Goutam et al., 2015; Ingvardsen et al., 2019; Li et al., 2018; Wang et al., 2023; Xie et al., 2021). In the actual production process, the traditional method of investigating wheat powdery mildew mainly relies on expert experience and manual field observations, which are time-consuming and laborious, are strongly subjective, and have low accuracy. Therefore, there is an urgent need to develop a fast and intelligent wheat powdery mildew segmentation method that is of great significance to guide the precise control of crops in the field and ensure food security.

In recent years, remote sensing technology has shown significant advantages in crop pest and disease monitoring, as it can monitor the occurrence of pests and diseases in real time and resolve many drawbacks of traditional manual methods. Pujari et al. (2014) used the support vector machine (SVM) algorithm to classify symptoms of wheat powdery mildew; the classification accuracy reached 90.83%. Guo et al. (2021) established a wheat stripe rust monitoring model based on partial least squares regression method; the monitoring accuracy was 0.75. Thangaiyan (2019) proposed a crop disease detection system segmentation method based on deformable models that segment features such as the color, gradient, texture, and shape of wheat leaf disease images. Xu et al. (2017) converted the wheat leaf rust into G single-channel gray image in RGB model and conducted vertical edge detection based on sober algorithm. Zhao et al. (2020) proposed machine learning algorithm based on hyperspectral image to achieve quantitative identification of wheat powdery mildew severity. An et al. (2024) proposed a method for classifying the degree of wheat powdery mildew infection by combining cascaded interval selection-sensitive bands with support vector machines. The abovementioned study is a common method for lesion segmentation and classification in traditional method. When the lesion area is similar to the background color or has blurred boundaries, poor segmentation results can follow. Therefore, the key to this research is to accurately segment diseased wheat leaves and spots under complex lighting and background condition in the field.

At present, with the rapid development of computer vision technology, various neural networks have been used in the automatic detection of crop disease image, and deep learning has strong feature extraction and pattern recognition capability, which is used to solve the problem existing in traditional crop detection method. To address the limitation of traditional image segmentation in terms of scene and task requirement, Zhang et al. (2019) used fully convolutional neural network and pulse-coupled neural network to quickly and accurately identify and classify wheat wilt disease grade. Su et al. (2020) proposed a wheat yellow rust recognition framework that combined the semantic segmentation model U-Net, drone multispectral, and RGB images. Zhang et al. (2024) proposed spore segmentation model of wheat scab CRF-ResUNet++; the MIOU reached 0.943. Li et al. (2024) proposed an improved Res-UNet method using attention-guided and scale-aware strategies. Liang and Wang (2020) proposed a framework based on improved U-Net, which has better segmentation performance. Liu et al. (2019) adopted the case-based transfer learning method to improve the accuracy of wheat powdery mildew monitoring in the field. Long et al. (2023) captured wheat disease images under field conditions and used the CerealConv model to segment powdery mildew; the accuracy rate is 97.05%. Mao et al. (2024) proposed a novel DAE-Ask method based on diversified enhanced features and edge features for wheat powdery mildew; the average detection accuracy rate is 96.02%. Önler and Köycü (2024) designed a model for detecting wheat powdery mildew using digital images. Multiple YOLOv8 model was trained using a collected custom image dataset, and the YOLOv8m model showed the highest accuracy, recall, F1, and average accuracy values of 0.79, 0.74, 0.770, 0.76, and 0.35, respectively. Shin et al. (2021) used AlexNet, SqueezeNet, GoogLeNet, ResNet-50, SqueezeNet-MOD1, and SqueezeNet-MOD2 to detect strawberry leaf powdery mildew; the classification accuracy is 95.59%. The abovementioned research can extract the feature of wheat leaf disease image at a deeper level. However, they cannot fully utilize the rich contextual information of wheat leaf images, and problems such as detail loss and blurred boundaries remain.

Transformer is a model based on attention mechanism, and the U-shaped network is a convolutional neural network architecture used for image segmentation. The combination of transformer and U-shaped network has become a research hotspot in the field of image segmentation and can improve the expressiveness and generalization performance of the model. Swin-Unet (Cao et al., 2021) effectively solves the problems of excessive computational load and unclear object-edge segmentation in U-shaped networks when processing large-scale images using a transformer. Zhang et al. (2023) proposed a weed recognition model based on improved Swin-Unet, which exhibited good segmentation performance. Yao and Jin (2022) improved the Swin-Unet model and successfully applied it to multiclass segmentation tasks of medium resolution remote sensing image, and achieved good performance.

In order to solve the problem of complex morphology, fuzzy boundary between diseased spots and non-diseased spots, and low segmentation accuracy, a segmentation method based on RSE-Swin Unet was proposed in this study. The method is based on Swin-Unet architecture and combines the ResNet and SENet modules to solve the problem. The main contributions of this study are to (1) establish a self-built image dataset for wheat powdery mildew, (2) introduce ResNet and SENet to enhance the feature extraction capability of powdery mildew, (3) construct an RSE-Swin Unet segmentation method using the Swin-Unet network, and (4) analyze the RSE-Swin Unet segmentation method and mainstream segmentation methods.

## Materials and methods

### Dataset preparation

**Figure 1:**
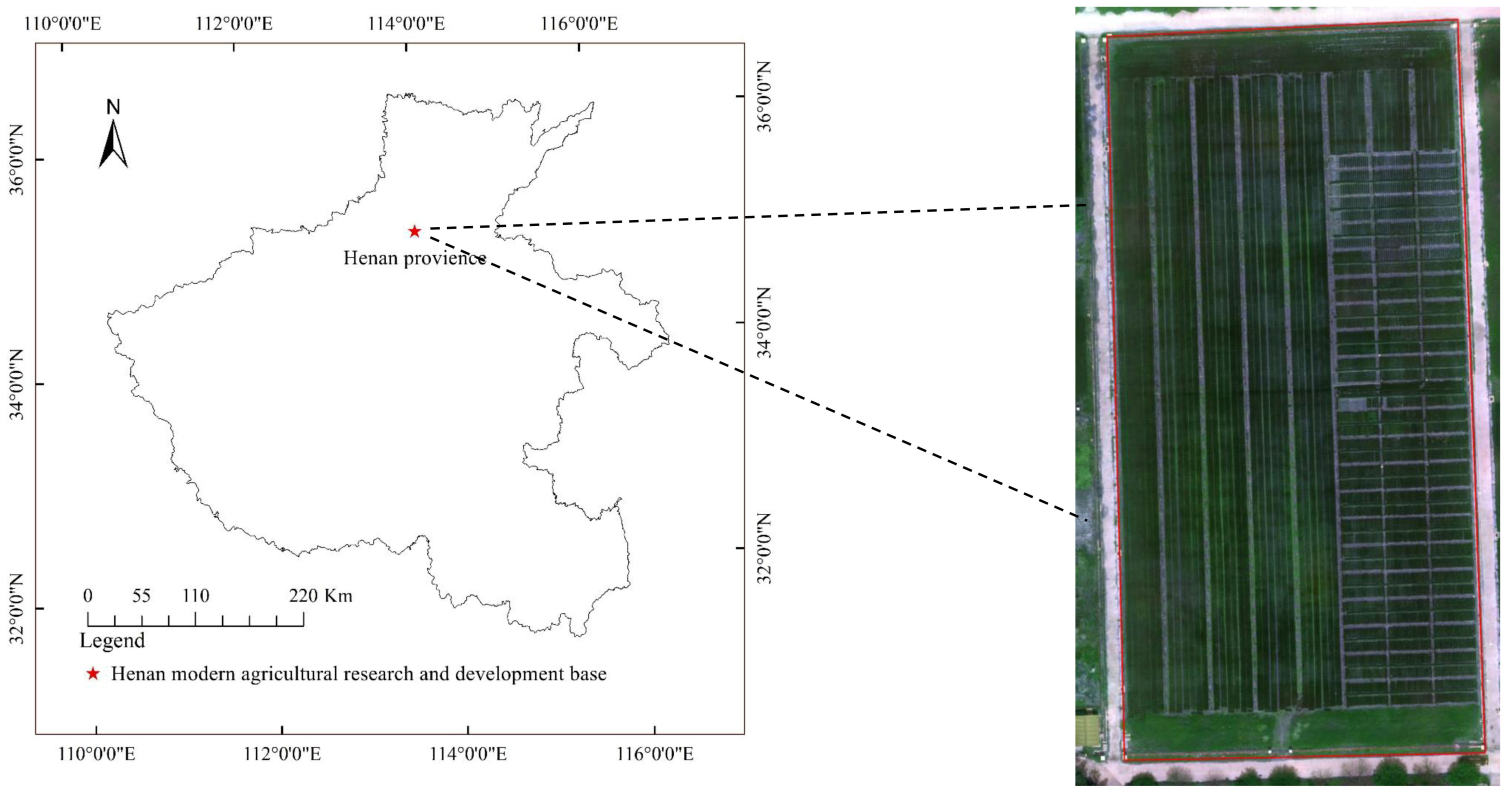
Location of the study area.

The experimental site is located at the Henan Modern Agriculture Research and Development Base of Henan Academy of Agricultural Sciences for wheat disease resistance identification, at 35°0′22″ N and 113°41′22″ E, as shown in **Figure 1**. The wheat powdery mildew in the study area was tested by artificial inoculation; therefore, the identification of wheat powdery mildew has a certain universality. Once wheat powdery mildew occurs on the leaves of different wheat varieties, the symptoms and signs of the affected areas are basically consistent.

**Figure 2:**
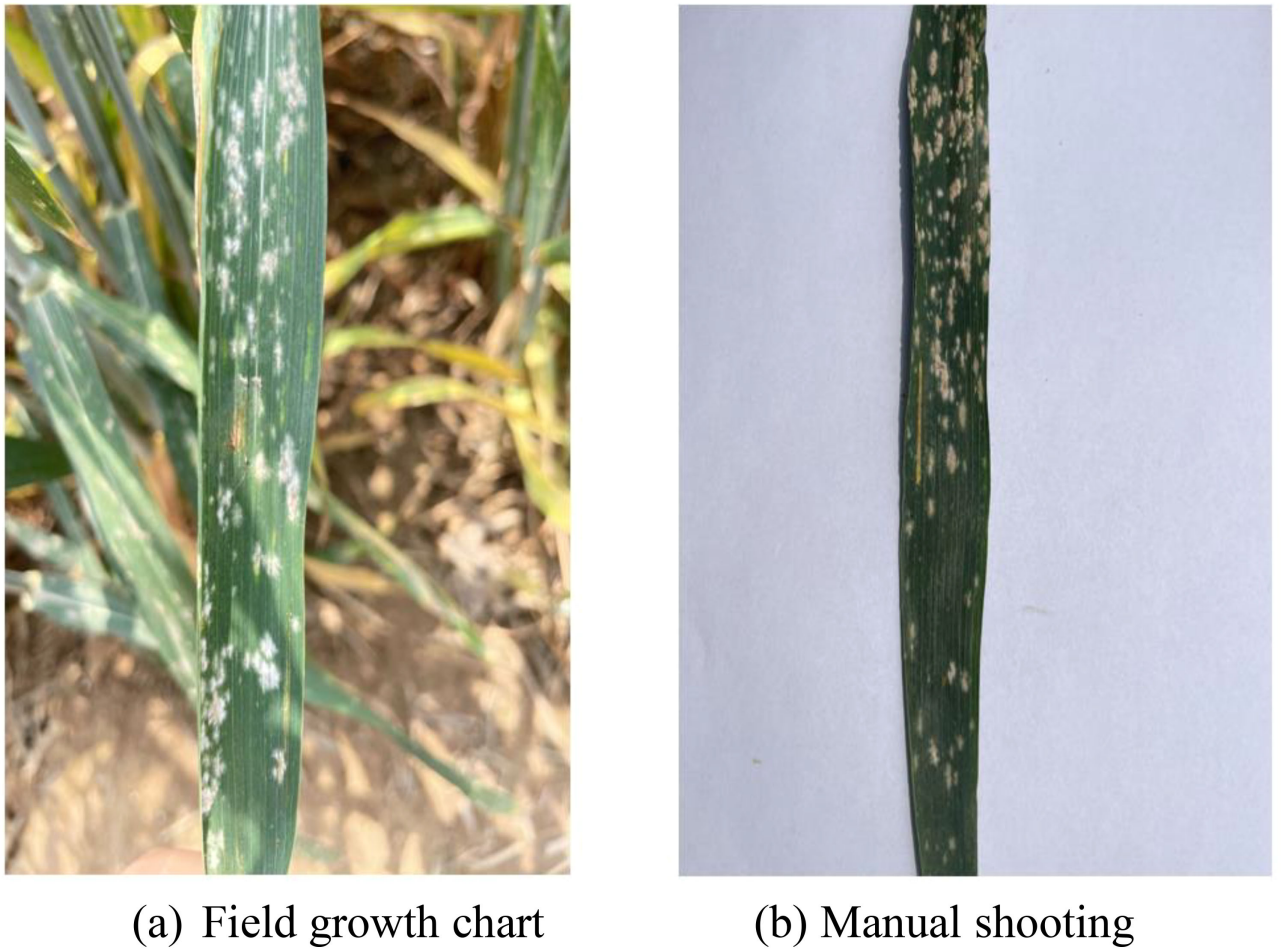
Example image of wheat powdery mildew. **(a)** Field growth chart **(b)** Manual shooting.

The field experiment adopted a completely randomized block design and was planted on October 23, 2023. There were 1,400 wheat varieties, and each plot area was 0.5 m^2^. Clear weather was selected for image capture using a smartphone (iPhone 12) with 12 million pixels and a resolution of 2,532 × 1,170. Equipped with A14BIonic chip, it supports optical image stabilization function. Images of wheat powdery mildew were collected from April 18 to May 2, 2023 and then every 5 days during the heading and flowering stages. The main focus is on capturing the wheat powdery mildew area, with a distance of 10 cm between the shooting equipment and the target. A total of 900 images were captured, with the resolution of each image being 4,032 pixels × 3,024 pixels. **Figure 2a** shows the field growth of wheat powdery mildew, while **Figure 2b** is a manually captured image.

Labelme labeling software (Russell et al., 2008) was used to label wheat powdery mildew images. Firstly, the “Create Polygon” tool was used to annotate the leaf category and then the spore category and the remaining parts were assigned to the background category. The segmentation task was carried out to divide the image into three categories: background, leaf, and spore.

### Dataset preprocessing

A total of 800 images were selected from the collected wheat powdery mildew images as the dataset and named as the wheat powdery mildew segmentation dataset. Deep learning typically requires a large amount of data to train a high-performance model. The use of data augmentation technique to expand the original dataset aims to effectively suppress model overfitting and improve the model’s generalization ability. The main method used for data augmentation include image cropping, image filtering, and color jitter.

### Image cropping

Image cropping is a widely used data augmentation method. The core purpose is to effectively increase the diversity of training data by capturing partial regions of the original image. Taking the segmentation task of wheat powdery mildew as an example, the position of the disease spot on the leaf is not fixed and unchanging. Through image cropping, the model can learn the features and local structure of the disease spot at various positions, thereby improving the segmentation ability of the model. Since the original image size was 4,032 pixels × 3,024 pixels, if you adjust it directly to 512 pixels × 512 pixels, the image will be distorted and a lot of information will be lost, making it difficult to distinguish between spores and spore edges, with a serious impact on subsequent analysis and handling. In view of this, dividing the original image into multiple 512 × 512 pixel-small images ensures that the semantic information in the image can be fully learned by the neural network. At the same time, dividing an image into several local images of 512 pixels × 512 pixels can also increase the amount of data. In the end, the 800 images were split into 3,000 smaller 512 × 512-pixel images.

### Image filtering

In order to effectively improve the generalization ability of the model, these pure background images are removed to ensure that the model can focus on learning image information containing the target. In raw images, since the percentage of targets is usually less than 100%, this means that not all raw images cover the target pixels. Therefore, after the segmentation of the image, a considerable proportion of the image will be entirely background image. In order to improve the generalization ability of the model, 1,240 pure background images were deleted, reducing the total number of images from 3,000 to 1,760.

### Color jitter

To enhance the robustness of the model, this study used techniques such as flipping, rotating, translating, and scaling to strengthen the dataset, reducing overfitting and improving the generalization ability. In addition, features extracted from hue, saturation, and HSV color space conversion yield superior results to help the model learn more diverse lesion features, thereby improving the model’s segmentation ability for different lesion features. The image and label are randomly flipped and rotated in the interval range of [-20°, 20°]. In the HSV color gamut, the hue channel varies randomly within the offset range [-180, 180], while the saturation and the offset of the HSV channel fall within the range of [-255, 255].

After data augmentation, a total of 3,000 images were obtained to address the issues of uneven and unbalanced data distribution. The dataset is divided into train set, validation set, and testing set in a ratio of 8:1:1 for subsequent network training. When processing high-resolution data, the large size of the original image data may lead to a significant consumption of memory and computing resources, resulting in extremely slow training speed and significantly increased training time for deep learning models. To solve this problem, we use data cropping in the preprocessing stage to unify all images to the same size. In practical applications, images can be adjusted to a fixed size through scaling or cropping operations to ensure consistency in model input. To avoid losing key image information, center cropping or random cropping is usually used to preserve important areas of the image. In addition, the distribution of images of different categories in the dataset is uneven, and the number of samples for certain lesions is relatively small, which may lead to the model tending to predict categories with a higher frequency of occurrence during training. To alleviate this problem, we adopt methods such as data augmentation to expand the diversity of data and enhance the model’s learning ability for various types of samples.

### Wheat stripe rust dataset

The image data of wheat stripe rust disease is provided by a publicly available dataset on Baidu Netdisk (data source: https://pan.baidu.com/s/1hYmyJxyFsWQpMro6Fix0ug?pwd=fp0k). This dataset contains 2,353 images of 538 different resolutions, with each image having a resolution of 512 pixels × 512 pixels.

### Deep learning method

**Figure 3:**
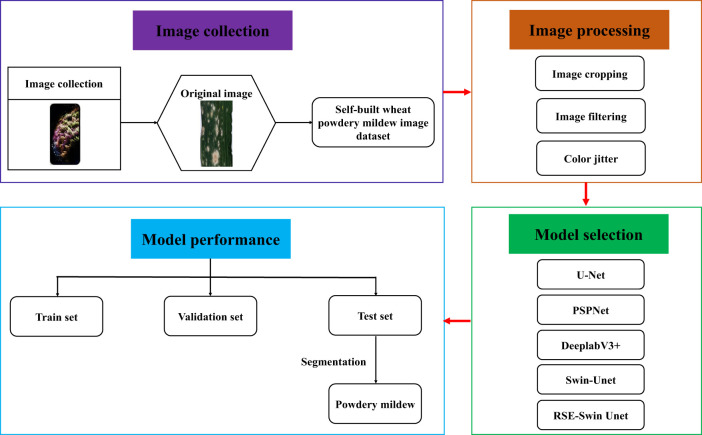
Framework for segmentation wheat powdery mildew using different deep learning methods.

The basic principle of deep learning method is built through a convolution layer, a pooling layer, and a fully connected layer, which is used for automatic feature extraction and classification of input data (Weng and Zhu, 2021). The convolution layer, as the base layer, is mainly responsible for the task of extracting image local features. The pooling layer is placed after the convolution layer to optimize the spatial proportion of the feature map. As the last layer, the fully connected layer classifies the features extracted by the convolution layer and the pooling layer. In this study, four mainstream deep learning methods were selected, namely, U-Net, PSPNet, DeepLabV3+, and Swin-Unet. All of these methods have been pretrained using transfer learning, as shown in **Figure 3**.

U-Net is the most common fully convolutional neural network model, and its excellent performance and simple structure make it one of the mainstream semantic segmentation models currently available (Weng and Zhu, 2021). It has a good segmentation performance and requires less computing power; however, owing to its overly simple network structure, there are some limitations. PSPNet is based on convolutional neural networks and extracts features through multiple convolutional layers, effectively utilizing global contextual information and adapting it to targets of different scales (Yuan et al., 2022). The core concept is to capture the feature information of different receptive fields using pyramidal pooling operations. DeepLabv3+ is a deep learning model used for semantic segmentation tasks that introduce techniques such as fully convolutional networks and dilated convolutions based on DeepLabv3 to improve the performance of semantic segmentation (Wang et al., 2022). Swin-Unet is the most representative semantic segmentation model based on a pure transformer (Cao et al., 2021); it uses the shift window mechanism proposed by Swin-Transformer to hierarchically extract features (Liu et al., 2021), greatly reducing the quadratic complexity of traditional self-attention mechanism and achieving better performance.

### Research methods

#### Swin-Unet

The overall structure of Swin-Unet is composed of encoder, bottleneck, decoder, and skip connection. Swin-Unet is a semantic segmentation network based on Transformer structure, which combines Swin-Transformer and Unet to achieve significant performance improvement in image segmentation tasks. The patch merging layer and patch extension layer are developed in Swin-Unet. The patch merging layer has the ability to improve the feature dimension of the image, and the patch extension layer can realize the upsampling operation of the image. In addition, in the process of image coding and decoding, the network structure realizes the self-attention mechanism from local to global and performs pixel-level segmentation and prediction tasks for global features, thus preserving image features well and effectively avoiding the misclassification of wheat powdery mildew region.

#### RSE-Swin Unet

**Figure 4:**
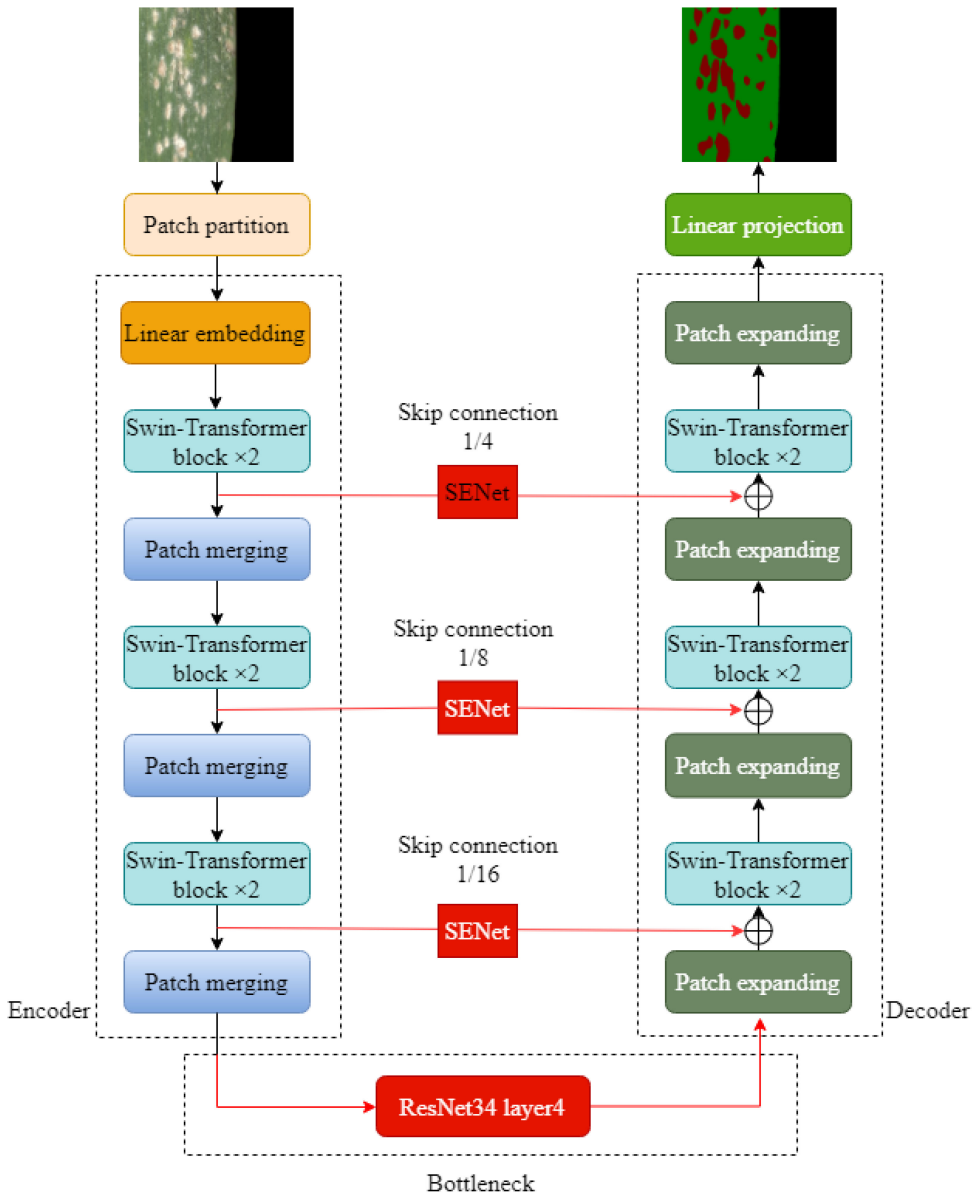
Network structure of improved Swin-Unet.

Aiming at the problems of complex morphology of wheat powdery mildew spots, blurred boundary between diseased spots and non-diseased spots, and low segmentation accuracy, this paper optimized and improved Swin-Unet and proposed wheat powdery mildew image segmentation method based on improved Swin-Unet, as shown in **Figure 4**. First of all, with Swin-Unet as the infrastructure and Swin-Transformer and SENet modules, the model can effectively capture global and local features in images and has strong feature expression capability. Secondly, the output of the SENet module is added to the corresponding feature tensor of the decoder to form the output feature tensor at the jump junction for subsequent decoder operations. Finally, in the deep bottleneck of Swin-Unet network, ResNet network layer is used to increase the extraction of sub-feature images and further enhance the segmentation effect of network features.

#### ResNet

ResNet is a deep neural network structure, which is widely used in computer vision tasks such as object detection and semantic segmentation (He et al., 2022). Because the residuals block in ResNet is designed so that it does not compromise the computational power of feature extraction, it is appropriate to replace two consecutive Transformer blocks at the bottleneck location with this residuals block. ResNet solves the degradation problem caused by network deepening through residual blocks. In order to better extract the regional characteristics of wheat powdery mildew and improve the overall segmentation effect, the bottleneck in Swin-Unet was improved in this study. After optimization and comparison, the layer4 structure in ResNet34 was used as the bottleneck in Swin-Unet to extract the depth feature of the lesion region in the bottleneck of wheat leaf image, which improved the segmentation accuracy of the model for wheat leaf lesion region.

**Figure 5:**
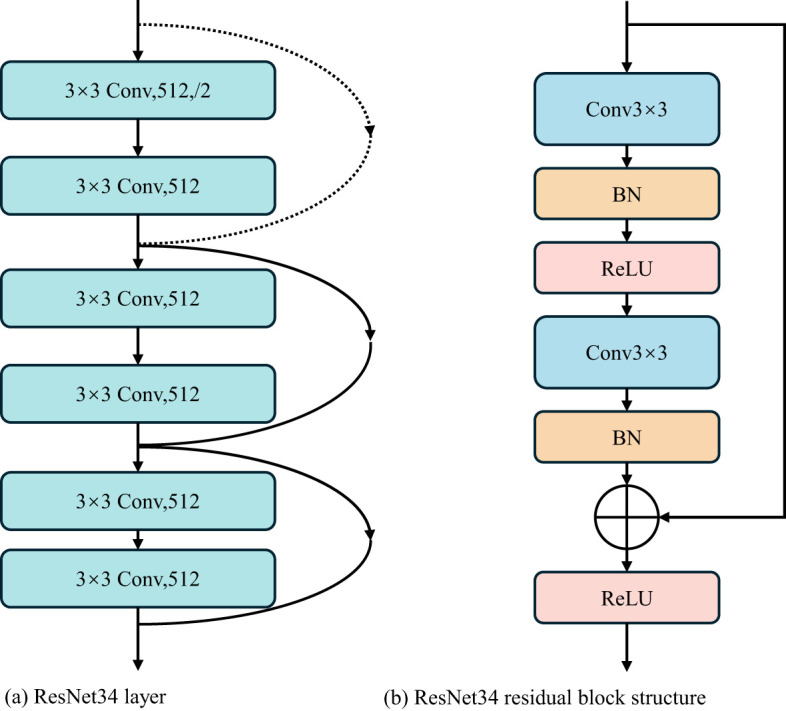
Structure of ResNet. **(a)** ResNet34 layer **(b)** ResNet34 residual block structure.

Using layer4 structure, the resolution and feature dimension of the image can be kept constant. The layer4 network structure of ResNet34 contained three residual blocks, as shown in **Figure 5a**. It can be seen that each residual module is mainly composed of two 3×3 convolutional layers, two-layer batch normalization (BN), residual block layer, and ReLU activation function, as shown in **Figure 5b**. The channel size of the shortcut layer of the side structure is twice the channel size of the residuals block. In order to make the number of channels before the last ReLU consistent, the channel size calculated in the residuals block and the shortcut is different. Through careful design of different channel sizes, deep convolution feature extraction is realized to ensure that the dimension remains unchanged.

#### SENet

**Figure 6:**
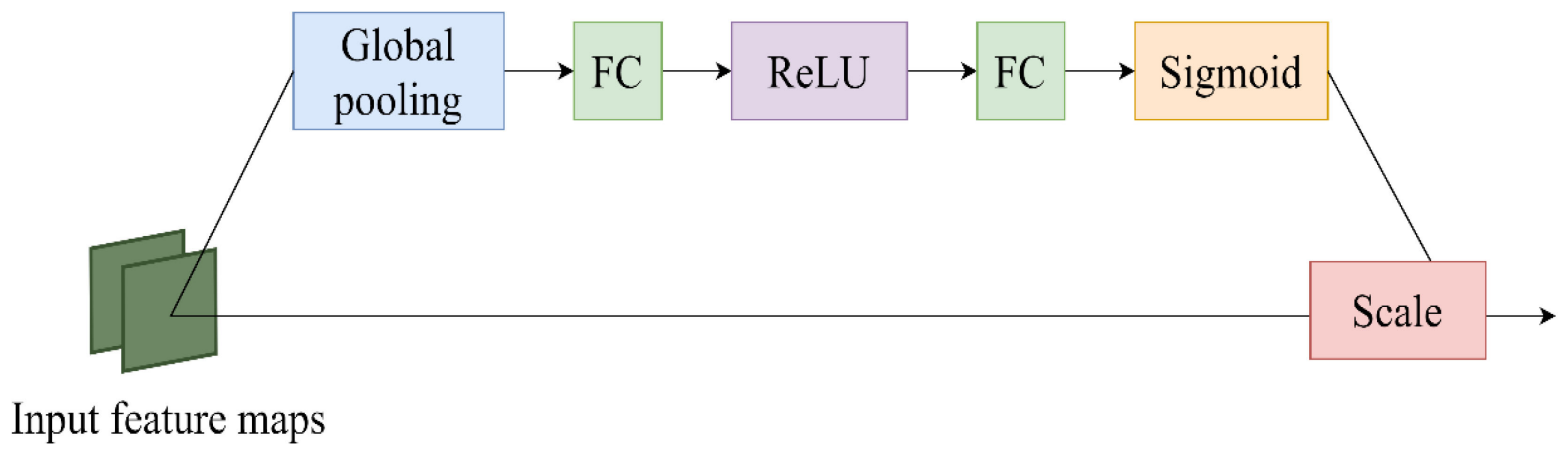
SENet module.

The core goal of the channel attention mechanism is the adaptive adjustment of channel feature weights so that the network can better focus on important features and effectively suppress relatively unimportant features. By learning the correlation between channels, SENet dynamically adjusts the weight of feature graphs to enhance the performance of important features and finally improve the performance of the model (Hu et al., 2020). The SENet structure is shown in **Figure 6**. In this study, the Swin-Unet network is improved, and the feature tensor of the jump connection is taken as the input content of SENet module every time a jump connection is made. Then, the Squeeze operation and the Excitation operation are applied to obtain the feature-weighted results. Finally, the output of the SENet module is added to the corresponding feature tensor of the decoder, forming the output feature tensor at the skip junction for subsequent decoder operations. This method greatly strengthens the close relationship between low-level and high-level features, thus providing more refined features for multi-scale prediction and segmentation.

### Loss function

In the task of image segmentation of wheat leaf powdery mildew, the disease area presents different sizes and shapes, and some intact wheat leaves may be marked as disease areas. The Dice loss function is more suitable for samples with class imbalance, but using this loss function can limit backpropagation and easily lead to gradient explosion, resulting in segmentation results that fail to achieve the expected results. In contrast, the BCE loss function is used in various classification tasks. In order to achieve better segmentation performance of wheat powdery mildew images and determine the most suitable loss function, this paper combines the Dice loss function with the BCE loss function. The calculation of the mixed loss function is as **Equation 1**.


(1) $$\begin{array}{c} {loss}_{total}=0.4*loss\_DICE+0.6*loss\text{\_BCE} \end{array}$$

In the mixed loss function, the weights of Dice and BCE loss functions are 0.4 and 0.6, respectively, determined based on the results of binary classification and empirical optimization of wheat leaf powdery mildew data in this paper. The weight of Dice loss is relatively low and has a relatively small impact on model optimization, while the weight of BCE loss is set to 0.6, which means that when calculating the overall loss, more attention is paid to the performance evaluation of binary classification tasks. Therefore, the mixed loss function can significantly improve the robustness and accuracy of wheat powdery mildew segmentation while controlling gradient stability.

### Experimental configuration

The experiment uses Intel (R) Core (TM) i7–10600 CPU, the main frequency is 2.90 GHz, the GPU selects NVIDIA GeForce RTX3090, the memory is 24 GB, and PyTorch is used as the deep learning framework. **Table 1** shows the hyperparameter settings used in the network model in this article, and all model parameters were trained, validated, and tested in a unified graphics card mode. The selection of learning rate is based on the grid search method implemented on the validation set, and the optimal initial value is determined through cross-validation, combined with the learning rate decay strategy to achieve accelerated convergence. The selection of batch size is based on the balance between model training stability and computational resources. We tried multiple values (4, 8, and 32) and ultimately chose 4 as the optimal value. The setting of the number of epochs is based on the performance of the validation set during the training process and is adjusted by monitoring the changes in the loss function to avoid the risk of overfitting. In terms of optimizer selection, we used the Adam optimizer to better adapt to the characteristics of the dataset.

**Table 1:** Hyperparameter settings for training.

Parameter	Parameter value	Configuration information
Epochs	100	Number of rounds of model training
Batch size	4	Number of training samples
Momentum factor	0.9	Methods to prevent model overfitting
Learning rate	0.0001	Adjust the parameters in the optimization algorithm
Input size	512 × 512	Size of the image input into the model

The batch size was four, momentum factor is 0.9, and the Adam optimizer was used for training. During the training and testing process, the number of training iterations amounts to 100, the initial learning rate is set at 0.01, and the learning rate decreases to 0.0001 as the number of iterations increased. The input network image is set to 512 pixels × 512 pixels.

### Model evaluation

To evaluate the performance of the model effectively and objectively, this paper employs accuracy (ACC), intersection over union (IoU), mean intersection over union (MIoU), mean pixel accuracy (mPA), precision, and recall as the evaluation indices of the semantic segmentation model. Using the abovementioned six evaluation indices to evaluate the accuracy of the wheat powdery mildew image segmentation results can reflect the segmentation accuracy more comprehensively. In this study, the number of parameters, computational energy (FLOPs), and model time were used to evaluate model performance. The higher the quantization value, the better is the effect of model segmentation. The calculation of IoU, precision, recall, F1, dice, accuracy, MIoU and mPA is seen with **Equations 2**–**9**.


(2) $$IoU=\frac{TP}{FP+FN+TP}$$

(3) $$Precision=\frac{TP}{TP+FP}$$

(4) $$Recall=\frac{TP}{TP+FN}$$

(5) $$\text{F}1=\frac{2\times \text{Precision}\times \text{Recall}}{\text{Precision}+\text{Recall}}$$

(6) $$MIoU=\frac{1}{k+1}\sum_{i=0}^{N}IoU$$

(7) $$\begin{array}{c} \text{Dice=}\frac{2\text{TP}}{2\text{TP+FP+FN}} \end{array}$$

(8) $$mPA=\frac{1}{k+1}\sum_{i=0}^{k}\frac{p_{ii}}{\sum_{j=0}^{k}p_{ij}}$$

(9) $$Accuracy=\frac{TP+TN}{TP+TN+FN+FP}$$

where TP (true positive) represents the sample whose median prediction result is positive and FP (false-positive) refers to the predicting positive and negative samples from the model. TN (true negative) means that the sample has a median predicted result that is negative. FN (false-negative) means that the model predicts a negative-positive sample. k+1 represents the total number of category. $p_{ij}$ represents the total number of pixels whose real pixel category is i predicted to be j . ${\text{p}}_{\text{ii}}$ represents the total number of pixels whose real pixel category is i predicted to be i .

## Results

### Training results of different segmentation methods

**Figure 7:**
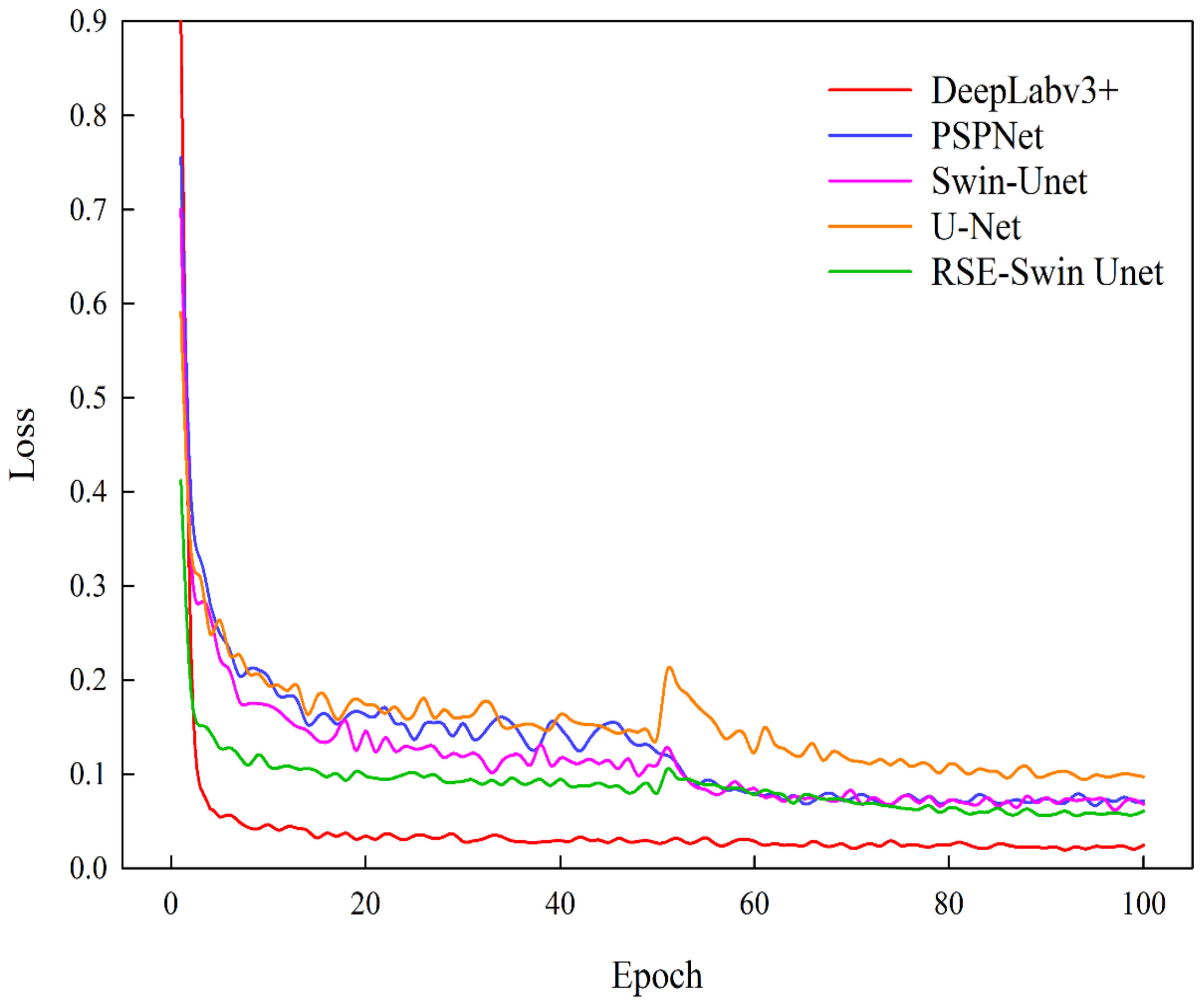
Comparison of training losses for different models.

Based on test sample data, the segmentation performance of DeepLabv3+, PSPNet, Swin-Unet, U-Net, and RSE-Swin Unet was compared. **Figure 7** shows the training loss curves of different segmentation models. As the number of iterations increases, the loss values of different segmentation methods decrease significantly at the beginning of training and gradually stabilize. Compared with other segmentation methods, the method proposed in this paper (RSE-Swin Unet) achieved the lowest loss value at the beginning of training and remained stable, proving that the method proposed in this paper is more efficient.

**Figure 8:**
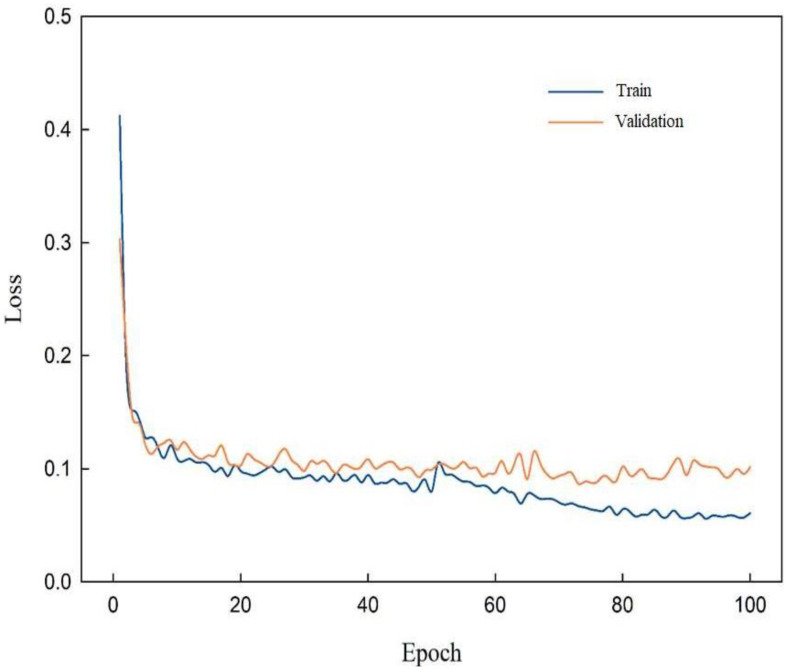
Training loss curve of RSE-Swin Unet.

Since Swin-Unet network loss feature information when processing multi-layer semantic information, SENet and ResNet are introduced to enhance the model’s ability to express powdery mildew feature, thereby achieving accurate segmentation of the model. To verify the superiority of RSE-Swin Unet, **Figure 8** shows the training loss curve of RSE-Swin Unet. In the early stage of network training iteration, the training loss curve of RSE-Swin Unet decreases rapidly. In the middle stage of training iteration, the training loss curve of RSE-Swin Unet maintains a moderate decrease. However, in the later stage of training iteration, the training loss curve of RSE-Swin Unet tends to be stable. At the same time, it is verified that the gap between the validation loss curve and the training loss curve is very small, which indicates that RSE-Swin Unet has excellent stability.

### Comparison analysis of the performance of different segmentation methods on wheat powdery mildew background, spore, and leaf

To verify the segmentation effect of RSE-Swin Unet on wheat powdery mildew images in this study, the U-Net, PSPNet, DeeplabV3+, and Swin-Unet networks were selected for comparative experiments. The performance comparison of different network models on wheat powdery mildew segmentation results is shown in **Table 2**.

**Table 2:** Comparison analysis of the performance of background, spore, and leaf with different segmentation methods.

Class	Methods	IoU (%)	Precision (%)	Recall (%)	F1 (%)	Dice (%)
Background	U-Net PSPNet DeeplabV3+ Swin-Unet RSE-Swin Unet	99.74 95.76 95.40 96.81 99.76	99.86 98.40 99.55 98.38 99.86	99.88 97.28 99.86 98.93 99.90	99.87 97.84 99.70 98.65 99.88	99.87 97.83 97.65 98.38 99.88
Spore	U-Net PSPNet DeeplabV3+ Swin-Unet RSE-Swin Unet	62.47 52.39 50.72 61.28 64.50	79.35 76.32 73.04 78.15 84.96	71.75 61.56 59.71 67.35 74.09	75.36 68.15 65.71 72.35 79.15	76.90 68.76 67.30 75.99 78.42
Leaf	U-Net PSPNet DeeplabV3+ Swin-Unet RSE-Swin Unet	86.04 84.55 82.21 85.63 85.01	92.67 92.45 89.39 92.31 93.36	94.63 93.61 91.10 92.68 95.90	93.64 93.03 90.24 92.49 94.61	92.50 91.63 90.24 92.26 91.90

In the background classification, the RSE-Swin UNet has a better segmentation effect, with IoU, precision, recall, F1, and Dice of 99.76%, 99.86%, 99.90%, 99.88%, and 99.88%, respectively. Compared with Swin-Unet, RSE-Swin Unet increased IoU, precision, recall, F1, and Dice by 2.95%, 1.48%, 0.97%, 1.23%, and 1.5%, respectively.

In spore classification, the RSE-Swin Unet has a better segmentation effect, with IoU, precision, recall, F1, and Dice of 64.50%, 84.96%, 74.09%, 79.15%, and 78.42%, respectively. Compared with Swin-Unet, RSE-Swin Unet increases IoU, precision, recall, F1, and Dice by 2.22%, 6.81%, 6.74%, 6.80%, and 2.43%, respectively.

In the leaf classification, the RSE-Swin Unet has a better segmentation effect, with precision, recall, and F1 of 93.36%, 95.90%, and 94.61%, respectively. Compared with Swin-Unet, RSE-Swin Unet improves precision, recall, and F1 by 1.05%, 3.22%, and 2.12%, respectively. U-Net has the highest IoU of 86.04%, which is 1.49%, 3.83%, 0.41%, and 1.03% higher than PSPNet, DeepLabv3+, Swin-Unet, and RSE-Swin Unet, respectively.

In summary, when IoU exceeds 50%, it shows that the target area predicted by the method is highly coincident with the real labeled target area, and the predicted background area is highly coincident with the real labeled background area, which means that the method may accurately separate the target from the image. The test results are shown in **Table 1**. The IoU of PSPNet and DeepLabv3+ for the segmentation of spores is close to 50%, indicating that the method has a poor effect on the segmentation of lesions, and there are problems of over-segmentation and background noise.

### Comparison analysis of the overall performance of different segmentation methods on wheat powdery mildew

To evaluate the superiority of the RSE-Swin Unet, the overall segmentation performance of wheat powdery mildew is shown in **Table 3**. RSE-Swin UNet has the best segmentation results and can accurately identify and segment lesions. MIoU, MPA, and accuracy are 84.01%, 89.96%, and 94.20%, respectively. Compared with Swin-Unet, MIoU, MPA, and accuracy increased by 2.77%, 3.64%, and 2.89%, respectively.

**Table 3:** Comparison analysis of the overall performance with different network methods.

Methods	Accuracy (%)	MIoU (%)	mPA (%)
U-Net	92.75	82.75	88.75
PSPNet	89.38	77.57	83.15
DeeplabV3+	88.58	76.11	82.89
Swin-Unet	91.31	81.24	86.32
RSE-Swin Unet	94.20	84.01	89.96

**Figure 9:**
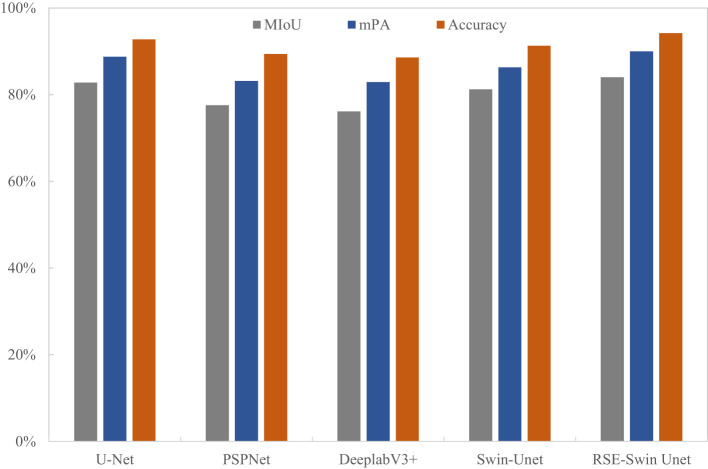
Visual representation of different methods for segmenting wheat powdery mildew image.

**Figure 9** shows the visual representation of different methods for segmenting the image of wheat powdery mildew. It can be found that compared with other methods, RSE-Swin Unet has the highest MIOU, mPA, and accuracy, which indicates that the introduction of ResNet and SENet can enhance the ability of feature extraction for wheat powdery mildew image, thereby improving image segmentation performance. This confirms that RSE-Swin Unet exhibits superior performance in wheat powdery mildew image segmentation.

### Comparison analysis of parameters, FLOPS, and model time of different segmentation methods on wheat powdery mildew

**Table 4** presents the comparison of different methods in parameters, FLOPs, and time. The parameters of PSPNet and FLOPS are the lowest, and the average segmentation time is shorter; however, the segmentation accuracy is low. Although the parameters, FLOPS, and average segmentation time of the RSE-Swin Unet are higher than other network models, the segmentation accuracy of RSE-Swin Unet is higher.

**Table 4:** Comparison analysis of parameters, FLOPS, and model time of different segmentation methods.

Methods	Parameters (M)	FLOPs (G)	Time (ms)
U-Net	43.93	184.00	19.5
PSPNet	2.38	5.87	6.10
DeeplabV3+	54.71	166.19	13.6
Swin-Unet	120.96	360.25	27.2
RSE-Swin Unet	140.58	451.97	36.4

### Visualization of wheat powdery mildew with different segmentation methods

**Figure 10:**
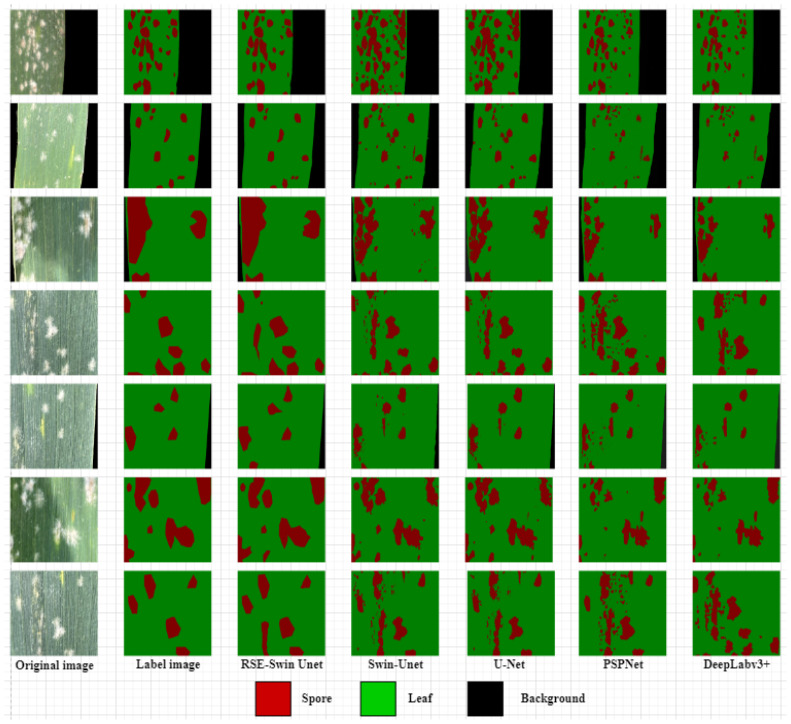
Comparison of the results of different segmentation methods.

To demonstrate the superiority of the proposed RSE-Swin Unet network structure in wheat powdery mildew image segmentation tasks, the improved RSE-Swin Unet was compared with the U-Net, PSPNet, DeeplabV3+, and Swin-Unet for segmentation results. The comparison of the segmentation results from different methods is shown in **Figure 10**. The results showed that RSE-Swin Unet achieved the best segmentation results in the mainstream segmentation methods because RSE-Swin Unet had good prediction results. Other mainstream segmentation methods can roughly segment lesion areas but cannot effectively segment adhesive and true lesion area. In this study, layer4 of ResNet34 is used as the bottleneck of the method, which increases the convergence of image feature calculation and improves the segmentation performance. Compared with Swin-Unet, RSE-Swin Unet has a higher segmentation accuracy. The comparative experimental results show that the RSE-Swin Unet combined with the Swin-Transformer and SENet modules is the best in terms of the segmentation results.

### Ablation experiment

To verify the effectiveness of RSE-Swin Unet, an ablation experiment was performed on different modules. The experimental results are listed in **Table 5**. By introducing the SENet module, the expression ability of wheat powdery mildew characteristics can be enhanced in complex backgrounds, which can effectively improve the performance of the model. By using the ResNet module and adding sub-feature image extraction, the segmentation accuracy of the model for wheat powdery mildew lesion areas has been improved. The addition of SENet module and ResNet module both contribute to improving the performance of the model. According to **Table 5**, introducing the SENet module and ResNet module separately resulted in network accuracies of 92.28% and 92.85%, respectively, which were 0.97% and 1.54% higher than Swin-Unet. Under the combined action of the abovementioned two improvement strategies, the final improved network achieved maximum improvement in accuracy, reaching 94.20%. Therefore, RSE Swin-Unet exhibits a strong segmentation performance.

**Table 5:** Results of the ablation experiment.

Methods	ResNet	SENet	Accuracy (%)	MIoU (%)	mPA (%)
Swin-Unet			91.31	81.24	86.32
Swin-Unet+SENet		✓	92.28	82.35	88.13
Swin-Unet+ResNet	✓		92.85	83.10	88.95
RES-Swin Unet+SENet+ResNet	✓	✓	94.20	84.01	89.96

### Generalization of different segmentation methods

**Figure 11:**
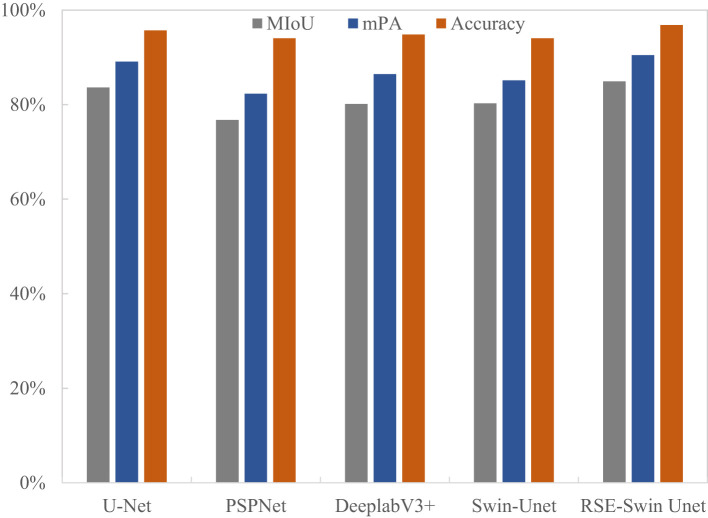
Visual representation of different methods for segmenting wheat stripe rust image.

**Figure 11** shows the overall segmentation performance of different segmentation methods for wheat stripe rust. It can be seen that RSE- Swin Unet has the best segmentation results, with accuracy, MIoU, and mPA of 96.88%, 84.91%, and 90.50%, respectively. Compared with Swin-Unet, it has improved by 2.84%, 4.64%, and 5.38%, respectively. This indicates that the method proposed in this study can accurately identify and segment wheat stripe rust lesions and is suitable for wheat stripe rust image segmentation.

## Discussion

From the aforementioned experimental outcome, it is evident that this study chose complex data for the wheat powdery mildew segmentation experiment, compared the mainstream segmentation methods with the method proposed in this study, and attained outstanding segmentation results in the processing of powdery mildew datasets. Deep learning can effectively segment the background of wheat diseases, spores, and leaves. However, for the original method, when the image background has high complexity, low contrast, and adhesion phenomena, the original network performs poorly in extracting target features, which is prone to cause the problem of missed segmentation. The proposed method in this study has significant advantages in segmentation accuracy and can meet the fundamental requirement of real-time detection of wheat powdery mildew.

To further validate the feasibility of the proposed approach in addressing issues such as the intricate morphology of diseased spots, the indistinct boundary between diseased and non-diseased spots, and the segmentation accuracy, it was compared with several methods aimed at resolving these problems. Liang and Wang (2020) put forward a wheat powdery mildew spore image segmentation model based on the enhanced U-Net framework, which demonstrates superior segmentation performance. However, owing to the lack of samples in their datasets, the model was overfitted, and the generalization ability was not strong. The proposed method in this study incorporates the SENet attention mechanism, which can accurately capture the lesion area and edge information. Yang et al. (2024) proposed an improved Swin-Unet model for the segmentation of maize diseases and obtained 84.61% segmentation results on the public dataset MIoU. Although the performance has been improved to some extent, there are still problems as to the number of parameters and the amount of computation. In this study, Swin-Transformer and SENet are combined to improve the efficiency and reasoning speed of the method. Lin et al. (2019) proposed a cucumber powdery mildew segmentation model based on convolutional neural network; the average pixel accuracy reached 96.08%. Because the image is obtained in the automatic analysis platform of the cucumber fruit leaf phenotype rather than in the field and the dataset sample is insufficient, the method has certain limitations. This study built a dataset of wheat powdery mildew to solve the robustness problem of the model. Through a comparison of the abovementioned methods, the method proposed in this study has better segmentation results, which solves the problems of complex lesion morphology of wheat powdery mildew, blurred boundary between lesions and non-lesions, and low segmentation accuracy. The proposed RSE-Swin Unet method in this study can accurately segment wheat powdery mildew image in challenging situations and has good computer vision processing and performance evaluation effects in the segmentation results of wheat powdery mildew dataset, providing a feasible method for accurate segmentation of wheat powdery mildew. However, when processing high-resolution images, directly adjusting the pixel size of the image to a smaller size may lead to information loss and image quality degradation, especially for task that require preserving details and accuracy. As the image data is cropped, the entire image is too large, resulting in poor segmentation results. In addition, the main drawback of our proposed RSE-Swin Unet method is that it has a large number of parameters and FLOPs, resulting in slower training time compared to Swin-Unet.

Different segmentation models can roughly segment wheat powdery mildew areas but cannot effectively segment adhesive lesions and true lesion areas. Due to the lightweight nature of PSPNet and DeepLabv3+networks, they may not be able to learn some complex features well, resulting in insufficient extraction of image attribute features, especially the difficulty in distinguishing between spots and spores. U-Net, PSPNet, DeepLabv3+, and Swin Unet models all exhibit under-segmentation and over-segmentation as well as some noise. Although U-Net has some false positive areas in the segmentation structure and cannot distinguish between lesion areas and backgrounds, compared to PSPNet, DeepLabv3+, and Swin Unet models, U-Net can better segment details. Due to the addition of SENet attention mechanism in this model, it focuses more on the most useful image features while suppressing useless image feature information. Therefore, RSE Swin Unet can comprehensively obtain lesion area and spore information, with better robustness and discrimination ability.

Most of the images in the existing publicly available wheat disease datasets are collected indoors, with simple backgrounds and a lack of noise interference. The models trained on this basis have poor anti-interference and robustness, making it difficult to adapt to the complex practical environment of farmlands. Creating a sufficiently rich and comprehensive database of crop diseases requires not only a significant investment of manpower, material resources, and time costs but also human errors in the process of disease image acquisition and expert annotation. Most of the existing crop disease segmentation models are relatively complex, relying on powerful computing power and massive memory, making it difficult to run on low-performance edge devices, unable to monitor diseases in real time, and unable to meet practical needs. However, the lightweight models currently developed on a smaller scale and suitable for mobile devices suffer from slightly lower accuracy and insufficient robustness, which reduces the effectiveness of the models in practical applications.

Li et al. (2022) used deep learning to perform semantic segmentation on wheat stripe rust images, dividing difficult to distinguish spores and spots into different categories and accurately segmenting the background, leaves (including spots), and spores. However, their method cannot fully utilize the rich contextual information of wheat leaf images, and there are still problems such as loss of details and blurred boundaries. In contrast, the dataset used in this study contains a large number of images, so RSE-Swin Unet effectively addresses the issues of information loss and boundary blurring. This study uses Swin-Unet as the basic model and introduces ResNet34 and SENet modules for improvement. A wheat powdery mildew image segmentation method based on the improved Swin-Unet is proposed, which can effectively capture global and local feature information in powdery mildew images, enhance the expression ability of the model for powdery mildew disease features, and provide a new method for automatic detection of wheat powdery mildew in complex field environments.

This study focuses on the image feature of wheat leaf powdery mildew and conducts in-depth research on the segmentation effect of leaf lesions. The Swin-Unet method has been successfully improved; although the proposed method in this study has better segmentation performance compared to other methods, there are still some problems that need to be solved, including (1) mixing public and self-built datasets, using data augmentation methods to increase dataset size and image complexity, (2) addressing the issue of poor performance in processing high-resolution images—we propose introducing a multi-scale processing mechanism to enhance the model’s generalization ability toward high-resolution images, and (3) we need to optimize RSE-Swin Unet, increase the dataset, and study how to improve the efficiency and interpretability of the method for wheat powdery mildew in complex field environments.

## Conclusion

This study constructed a wheat powdery mildew dataset through a wheat disease resistance identification experiment. Based on Swin-Unet, an improved Swin-Unet image segmentation method for wheat powdery mildew was proposed. With the introduction of ResNet and SENet module, this method can effectively capture the global and local feature information of powdery mildew image and enhance the ability of the model to express the feature of powdery mildew. The test results showed that RSE-Swin Unet could quickly segment wheat powdery mildew. The MIoU, mPA, and accuracy values were 84.01%, 89.96%, and 94.20%, respectively, which were 2.77%, 3.64%, and 2.89% higher than the original Swin-Unet. At the same time, compared with other network methods, the proposed method has better computer vision processing effect and performance evaluation and detection effect, and the segmentation performance is the best. The method overcomes the problems of low segmentation accuracy caused by the complex shape of the lesion and the blurred boundary between the lesion and non-lesion in the image of wheat powdery mildew and can accurately segment wheat powdery mildew under a complex background, providing a new method for the automatic detection of wheat powdery mildew.

## Data Availability

The raw data supporting the conclusions of this article will be made available by the authors, without undue reservation.
